# Professor Barrett's Paper before the Institute of Heating and Ventilating Engineers

**Published:** 1905-07-22

**Authors:** 


### EDITOR'S LETTER-BOX.

### PROFESSOR BARRETT'S PAPER BEFORE THE INSTITUTE OF HEATING AND VENTILATING ENGINEERS.

THE ENCLOSED AND OPEN GAS BURNER.

SIR,—I shall make the best use of the space at my disposal by examining the problem of the enclosed and the open gas-burner. The heating efficiency of an ordinary gas flame is ridiculously small. This is seen when we remember that a 5-burner should liberate sufficient heat to raise the temperature of a room 20 ft. by 14 ft. by 10 ft. 1° F. per minute. All the conditions are unfavourable to efficient combustion. The zone of combustion is very small, leading immediately to a much colder medium; both gas and air arrive cold, and are driven rapidly through, while the very small surface gives rise to only very small convection currents; so that the distribution of what heat is generated is very bad. When the flame is enclosed, as described by Professor Barrett, all the conditions are changed, very largely in favour of efficient combustion. A hot combustion-chamber is provided, in which the products of combustion are detained for a comparatively long time, and whose hot outer walls give rise to powerful convection currents, while the gas and air arrive at a higher temperature. The result is, that a far larger percentage of the carbon and hydrogen present are oxidised, a larger quantity of heat being liberated, and the heat liberated is distributed far more efficiently. Radiation, by the Professor's own showing, can play only a very small part in the operation. There is no paradox about this. Every engineer who has studied the problem knows it, and several manufacturers have taken advantage of it to produce apparatus in which the ordinary gas-flame is employed to economically heat water and air. I criticised severely in my report, because the explanation given by the Professor illustrates the teaching too often supplied to engineering students in our technical colleges, the professoriate in very many cases having had no opportunity for testing their often crude theories in practice.—YOUR ENGINEERING CORRESPONDENT.

